# The dynamics of folding instability in a constrained Cosserat medium

**DOI:** 10.1098/rsta.2016.0159

**Published:** 2017-04-03

**Authors:** Panos A. Gourgiotis, Davide Bigoni

**Affiliations:** 1 School of Engineering and Computing Sciences, Durham University, South Road, Durham DH1 3LE, UK; 2 DICAM, University of Trento, via Mesiano 77, 38123 Trento, Italy

**Keywords:** complex media, wave propagation, patterning, Green’s function

## Abstract

Different from Cauchy elastic materials, generalized continua, and in particular constrained Cosserat materials, can be designed to possess extreme (near a failure of ellipticity) orthotropy properties and in this way to model folding in a three-dimensional solid. Following this approach, folding, which is a narrow zone of highly localized bending, spontaneously emerges as a deformation pattern occurring in a strongly anisotropic solid. How this peculiar pattern interacts with wave propagation in the time-harmonic domain is revealed through the derivation of an antiplane, infinite-body Green’s function, which opens the way to integral techniques for anisotropic constrained Cosserat continua. Viewed as a perturbing agent, the Green’s function shows that folding, emerging near a steadily pulsating source in the limit of failure of ellipticity, is transformed into a disturbance with wavefronts parallel to the folding itself. The results of the presented study introduce the possibility of exploiting constrained Cosserat solids for propagating waves in materials displaying origami patterns of deformation.

This article is part of the themed issue ‘Patterning through instabilities in complex media: theory and applications.’

## 1. Introduction

Folding is a mechanical process involving the formation of a narrow, highly curved element separating large zones of low curvature. Although this feature is only scarcely present in natural systems, it can have important technological applications connected to the realization of origami-inspired transformable materials [1]. A usual approach to folding is in terms of localization of bending in a post-bifurcation deformation pattern, a phenomenon involving large strain [2,3]. Another approach explains folding as induced by deformation of a generalized continuum working in proximity of a material instability threshold, namely failure of ellipticity [4–6]. The latter approach was developed for constrained Cosserat elastic materials with extreme orthotropy, by employing a quasi-static Green’s function as a perturbation to show that ellipticity loss induces stress channelling, folding and faulting. In this article, the acoustic tensor is derived, and wave propagation conditions are explored for anisotropic constrained Cosserat solids, with full account of microinertia. For these materials, a new infinite-body, time-harmonic Green’s function is obtained under antiplane deformation and used to explore the dynamical behaviour of an extremely orthotropic material prone to folding. Results show that folding localizes at the steadily pulsating force applied as a perturbation to a constrained Cosserat material with extreme orthotropy and waves emanate from the source that degenerate into plane disturbances parallel to the direction of ellipticity loss. Moreover, maps of displacements reveal the emergence of complex patterns of deformation, typical of the material instability of a Cosserat continuum. Special attention is devoted to the presence of rotational microinertia. This feature is explored as connected to pattern formation. It is shown that its magnitude can change the sign of the lower-order derivatives in the differential equations of motion, so that its effect on the emergence of deformation patterns is very complex and sometimes counterintuitive. In fact, as related to the presence of microinertia, the formation of a folding wave propagating along the discontinuity lines is shown to become possible.

## 2. Dynamics of couple-stress elasticity

In this section, the basic elastodynamic equations are introduced for linear anisotropic couple-stress solids. A detailed presentation of the couple-stress theory (called also ‘constrained Cosserat theory’) can be found in [7] (see also [4,8]).

The kinetic nergy density, evaluated with respect to an inertial frame of reference, differs from the classical form due to the presence of the microinertia of the continuum which measures, through the symmetric structural tensor 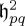, the effect of the spin 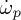. It can be written as [9], p. 248
2.1
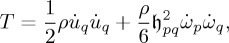
where *ρ*>0 is the mass density, *u*_*q*_ is the displacement vector, 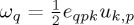 the rotation vector (*e*_*qpk*_ is the Levi-Civita alternating symbol) and the superposed dot denotes time differentiation. The components of the structural microinertia tensor 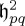 have the dimensions of a squared length. In the following, rectangular Cartesian coordinates are employed together with indicial notation and the usual summation convention on repeated indices.

The microinertia of the continuum introduces a more detailed description of motion in the present theory than in the Cauchy (or ‘classical’ in the following) theory.^1^ Nonetheless, as a particular case of the developed theory, the rotary microinertia tensor 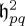 can be set equal to zero, so that in this simple case the spin does not play a role in the kinetic energy.

Employing the balance laws for linear and angular momentum, the local forms of the equations governing the dynamics of a constrained Cosserat medium are obtained [8,11]:
2.2
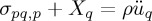
and
2.3
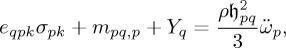
where *σ*_*pq*_ and *m*_*pq*_ denote the stress and couple-stress tensors (both asymmetric), and *X*_*q*_ and *Y*_*q*_ are, respectively, the body force and the body moment, both measured per unit volume.

Decomposing the stress tensor *σ*_*pq*_ into a symmetric *τ*_*pq*_ and antisymmetric *α*_*pq*_ part and using equation (2.3), the antisymmetric part of the stress tensor can be written as
2.4
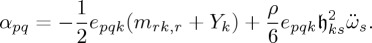
A combination of equations (2.2)–(2.4) yields a single equation of motion for the symmetric part of the stress tensor and the deviatoric part of the couple-stress tensor:
2.5
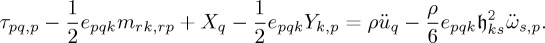


The traction boundary conditions at any point on a *smooth* boundary consist of the following *three reduced* force-tractions and *two tangential* couple-tractions [7,12]:
2.6
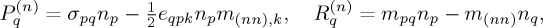
where *n*_*p*_ denotes the unit normal to the boundary, and *m*_(*nn*)_ is the normal component of the couple-stress tensor *m*_*pq*_, so that *m*_(*nn*)_=*m*_*pk*_*n*_*p*_*n*_*k*_.

For linear constitutive behaviour, the strain energy density assumes the following general quadratic form in the case of *centrosymmetric* materials:
2.7
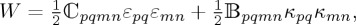
where *ε*_*pq*_ is the standard infinitesimal strain tensor and *κ*_*pq*_=*ω*_*q*,*p*_ is the curvature tensor (the transpose of the gradient of rotation), which by definition is traceless, *κ*_*pp*_=0. The elasticity tensors 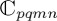 and 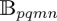 are equipped with the following symmetries: 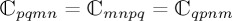, 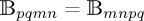 and 
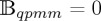
 (the last equality follows directly from the fact that the curvature tensor is purely deviatoric [7]). Therefore, in addition to the 21 independent constants defining the classical elasticity tensor 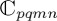, 36 independent constants are needed for defining 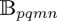. The corresponding constitutive equations are
2.8
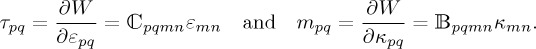
It is remarked that the Cauchy elastic behaviour is recovered when tensor 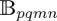, defining a ‘purely Cosserat behaviour’, is set to zero. The conditions for positive definiteness of the strain energy density, strong ellipticity of the elasticity tensors and the related van Hove uniqueness theorem were given recently in [4]. Moreover, assuming that the kinetic energy density is positive definite implies, in turn, that 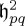 is also positive definite.

A substitution of the constitutive equations (2.8) into the governing equation (2.5) yields the equations of motion in terms of the displacements,
2.9
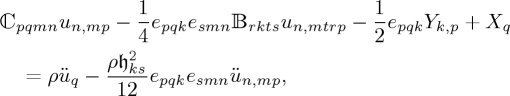
which represent the counterpart of the Navier–Cauchy equations in the classical theory.

## 3. The acoustic tensor and the propagation of plane waves

The propagation of plane harmonic waves is defined through a displacement field in the form
3.1
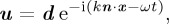
where i^2^=−1, *t* denotes time, ***d*** denotes the wave amplitude vector, ***n*** denotes the unit propagation vector and *k* denotes the wavenumber, in general complex. Moreover, vector ***x*** denotes the position vector, *ω* the angular frequency, taken to be real, and *V* =*ω*/*k* the phase velocity.

A substitution of equation (3.1) into the equations of motion (2.9), with null body forces and couples, leads to the propagation condition
3.2
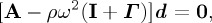
where **I** is the identity tensor, and
3.3
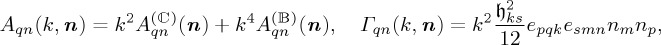
with
3.4
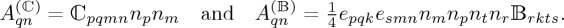
Note that the symmetries of the elasticity tensors 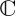 and 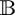 imply that 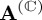 and 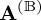 are symmetric second-order tensors, and thus **A** is also symmetric. In addition, the symmetry of tensor 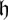 implies that ***Γ***=***Γ***^T^.

As shown in Gourgiotis & Bigoni [4], 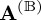 is a singular tensor that always possesses one null eigenvalue corresponding to the eigenvector ***n***, i.e. 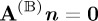. The same property is shared also by the tensor ***Γ***, which is related to the microinertia of the continuum. In fact, it can be readily shown that
3.5
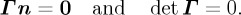
An immediate consequence of the properties of the tensor ***Γ*** is that, if 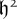 is *positive semi-definite*, the two (non-trivially null) eigenvalues of ***Γ*** are always non-negative. Under these circumstances, the tensor **I**+***Γ*** is *always positive definite*, and thus invertible. The latter observation enables us to recast equation (3.2) in the form
3.6
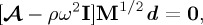
where
3.7
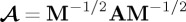
is the acoustic tensor for a constrained Cosserat medium with microinertia and **M**=**I**+***Γ***. Note that the acoustic tensor is symmetric. Furthermore, for a continuum without microinertia (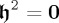), the acoustic tensor reduces to 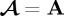, a case that has been thoroughly examined in [4].

A non-trivial solution to the eigenvalue problem (3.6) exists when
3.8
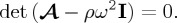
Condition (3.8) implies that, for plane waves to propagate with positive speed and for all real wavenumbers *k*, the eigenvalues *ω*^2^ (to within a multiplicative constant *ρ*) of the acoustic tensor 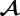 must be strictly positive. Sufficient conditions to ensure wave propagation (WP) in a constrained Cosserat medium with microinertia are that **A** is positive definite and 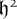 is positive semi-definite. The conditions for tensor **A** to be positive definite were given in [4]. It should be noted that, although the eigenvectors **M**^1/2^***d*** of the acoustic tensor in equation (3.6) are orthogonal, the corresponding motion vectors ***d*** are generally not.

In the light of the above discussion, the sufficient conditions for (WP) reduce then to the following inequalities:
3.9
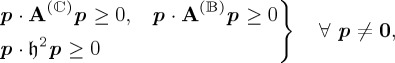
augmented with the condition ***p***⋅**A*p***≠0, so that both ‘=’ cannot simultaneously hold in the first two inequalities in equations (3.9). In other words, the above conditions imply that the vector ***p*** cannot be an eigenvector corresponding to a null eigenvalue of both the classical part 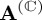 and the couple-stress part 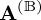 of the acoustic tensor. Note that, in the case where the wave amplitude vector ***d*** is parallel to the propagation vector ***n***, equation (3.2) degenerates to the classical condition
3.10
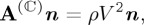
which implies that for every couple-stress anisotropy 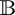 and microinertia anisotropy 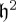 at least one direction of propagation exists such that the wave characteristics are governed only by the Cauchy elastic part of the constitutive equations. This direction coincides with the direction of propagation of purely longitudinal P-waves in a classical anisotropic medium (see also [4]).

## 4. Antiplane deformations for orthotropic couple-stress materials

In this section, the governing dynamical equations and various stability criteria are derived for an orthotropic couple-stress material, including microinertial effects under antiplane deformations. It is worth noting that the general three-dimensional quasi-static equations for an orthotropic couple-stress solid were given in [4]. Moreover, the elastodynamic equations for isotropic couple-stress materials under antiplane deformations can be found in [13,14].

### (a) Governing equations and positive definiteness conditions

For a body occupying a region in the (*x*,*y*)-plane under antiplane strain conditions, the displacement field assumes the following form:
4.1
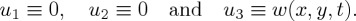
Accordingly, the non-vanishing components of strain, rotation and curvature are given as
4.2
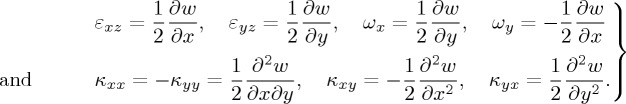
Further, considering an orthotropic centrosymmetric material and assuming that the axes of orthotropy coincide with the employed rectangular Cartesian system, the constitutive equations (2.8) reduce to [4]
4.3
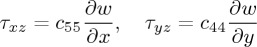
and
4.4
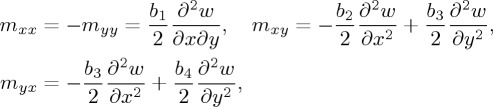
where *c*_44_ and *c*_55_ are the classical shear moduli characterizing an orthotropic Cauchy material subject to antiplane conditions, and *b*_*q*_ (*q*=1,…,4) are the couple-stress orthotropic moduli, with the dimension of a force (for a detailed discussion on orthotropic couple-stress materials, the reader is referred to appendix A in [4]). An inspection of the constitutive equations (4.4) reveals that there is a direct analogy between a constrained Cosserat material under antiplane deformation and an orthotropic Kirchhoff plate [15]. Indeed, the couple-stress components *m*_*xy*_ and *m*_*yx*_ may be identified with the bending moments, and *m*_*xx*_ and *m*_*yy*_ with the twisting moments, applied on an element of a plate. In this context, the couple-stress parameters *b*_2_/2 and *b*_4_/2 represent the bending stiffnesses in the principal *x*- and *y*-directions, *b*_1_/2 the principal twisting stiffness and *b*_3_/2 the stiffness associated with the effects of secondary bending (Poisson’s effect).

For a positive definite strain energy density (PD), the material moduli must satisfy the following inequalities:
4.5
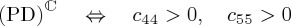
and
4.6
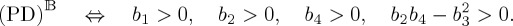
Moreover, the structural microinertia tensor 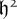, in the general orthotropic case, has three independent components 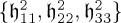, one for each of the principal axes of orthotropy. In view of equation (2.4) and assuming zero body couples, the antisymmetric components of the stress tensor become
4.7
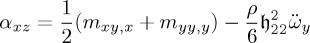
and
4.8
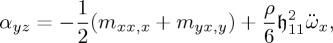
so that, taking into account equations (4.3), the shear stresses assume the following form in terms of the out-of-plane displacement:
4.9
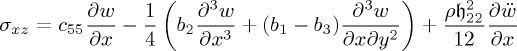
and
4.10
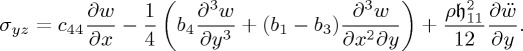
Note further that, for the kinetic energy density to be positive definite, the microinertia moduli must satisfy the following inequalities:
4.11
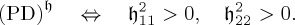
Finally, the equation of motion for the out-of-plane displacement becomes
4.12
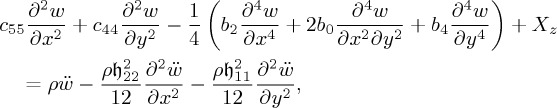
where *b*_0_=*b*_1_−*b*_3_ is a material parameter that accounts for both torsion and secondary bending effects.

In the case of material isotropy, the stiffness and inertia moduli become *c*_44_=*c*_55_=*μ*, *b*_1_=4*η*+4*η*′, *b*_2_=*b*_4_=4*η*, *b*_3_=4*η*′ and 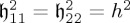, so that the equation of motion (4.12) reduces to
4.13
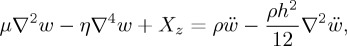
an equation which, in the absence of body forces, was given first by Clebsch [16], p. 797, equation (318a) to describe the motion of a plate, including prestress and rotational inertia effects.

### (b) Time-harmonic response and ellipticity conditions

According to the time-harmonic assumption, the displacement is represented as
4.14
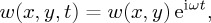
so that the equation of motion becomes now
4.15
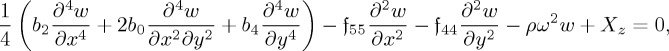
with
4.16
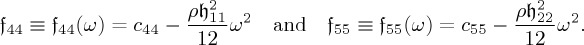
In order to classify the partial differential equation (4.15), one has to examine only the principal (fourth-order) part of the differential operator related with the Cosserat moduli *b*_*q*_ (see for instance Renardy & Rogers [17]). This implies that the classification of equation (4.15) for the time-harmonic response remains the same as for the quasi-static case. In fact, the latter case was examined in Gourgiotis & Bigoni [4], where the conditions of ellipticity (E) were explicitly derived. Here the elliptic regime only is considered, defined through the following conditions [4]:
4.17
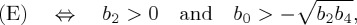
holding for *b*_4_>0 (the bending stiffness in the *y*-direction is assumed to be always positive). In particular, two regimes of ellipticity (E) can be distinguished:

(i) the elliptic imaginary (EI) regime for *b*_2_>0, 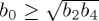; and
(ii) the elliptic complex (EC) regime for *b*_2_>0 and 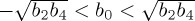.

The emergence of weakly discontinuous surfaces corresponds to failure of ellipticity, as in the quasi-static case [4]. This occurs in a continuous loading path (starting from (E)) either when 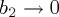 with *b*_0_>0 or when 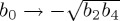 with *b*_2_>0. The former case defines the elliptic imaginary/ parabolic (EI/P) boundary, and the latter the elliptic complex/hyperbolic (EC/H) boundary. In both cases, the material exhibits an extreme orthotropic behaviour.

It is further remarked that failure of (PD) and the related loss of uniqueness for a boundary value problem of antiplane deformation can arise simultaneously with loss of (E). Indeed, according to equations (4.6) and (4.17), this situation occurs in a Cosserat material for which: (i) 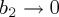 and 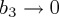, so that ellipticity is lost at the (EI/P) boundary, or (ii) 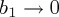 and 
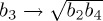
, so that ellipticity is lost at the (EC/H) boundary. Therefore, a material can be designed to work in antiplane strain conditions and display extreme behaviours (such as stress channelling and emergence of localized folding), but still preserving uniqueness of the solution.

Finally, it is interesting to observe that the terms 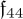 and 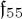 in equation (4.15) are related to the lower-order part of the differential operator and may change sign according to the values of the microinertia parameters (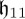, 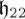). Indeed, for a fixed frequency *ω*, these terms could become negative for high values of (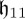, 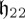), which, in turn, implies that, although the equation remains elliptic, the solution would change character. From the viewpoint of plate theory, such a change of sign would correspond to passing from tensile to compressive prestress in the *x*- and *y*-directions.

### (c) SH waves in an orthotropic medium

Antiplane shear (or SH) motions are now examined in a homogeneous orthotropic constrained Cosserat medium with microinertia. Assuming zero body forces and substituting into the equation of motion (4.12) a plane-wave harmonic solution of the form
4.18
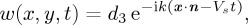
the dispersion equation is obtained, relating the phase velocity *V*_*s*_ of SH waves to the wavenumber *k* as
4.19
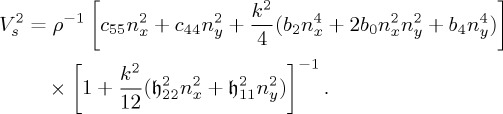
For an orthotropic material under antiplane motions, the only non-vanishing component of the acoustic tensor is
4.20
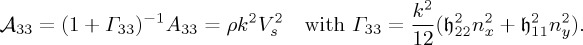
The (WP) condition requires that SH waves propagate with real non-zero velocities for all real wavenumbers *k* in any direction ***n***, which means that 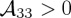. In view of the inequalities (3.9), the (WP) condition can then be defined as
4.21
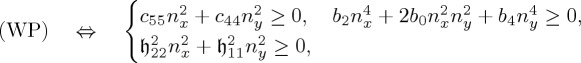
augmented with the condition *A*_33_≠0, so that the ‘=’ in the first two inequalities in (4.21) cannot hold simultaneously. In particular, for the first and the third inequalities to hold, the shear moduli (*c*_44_, *c*_55_) and the microinertia parameters (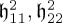) must be non-negative. Moreover, the second inequality requires that *b*_2_≥0 and 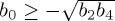.

It is worth noting that, for a constrained Cosserat material, waves can propagate while ellipticity is lost. For example, in the special case of an extreme orthotropic couple-stress material with *b*_2_=0 and *b*_0_>0, the condition (E) fails, but SH waves may still propagate for all wavenumbers and in all directions, provided that *c*_44_≥0 and *c*_55_>0. This is in marked contrast with the classical elasticity case, where loss of (E) would imply violation of the (WP) condition. Indeed, it is recalled that the (E) condition in the classical elasticity case requires that *c*_44_≠0 and *c*_55_≠0, whereas the (WP) condition implies that *c*_44_>0 and *c*_55_>0.

Finally, it remarked that, setting one or both the microinertia parameters to zero, SH waves can still propagate. Nonetheless, microinertia plays an important role, because for large wavenumbers, 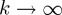, the phase velocity remains bounded and attains the constant value
4.22
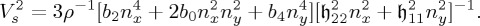
The finiteness of the phase velocity for large wavenumbers is in agreement with the results for the classical structural models of a Rayleigh beam and a Love rod [18,19]. Note further that in the special case where *b*_2_=0, so that (E) is lost at the (EI/P) boundary, the phase velocity (4.19) of a wave propagating in the direction ***n***=(±1,0) becomes inversely proportional to the wavenumber *k*. The latter observation suggests that the presence of microinertia will cause high-frequency (large-wavenumber) disturbances to ‘almost’ stop propagating in this direction. In fact, in this direction the medium behaves as a Cauchy material without Cosserat effects but with non-zero microinertia, having a phase velocity of the form 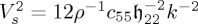. An analogous conclusion is reached in the case where (E) is lost at the (EC/H) boundary.

### (d) Time-harmonic Green’s function

A time-harmonic concentrated body force *X*_*z*_=*Sδ*(*x*)*δ*(*y*) e^i*ωt*^ is applied in an infinite orthotropic constrained Cosserat material with microinertia subjected to antiplane deformation. This problem set-up allows one to determine the Green’s function for the out-of-plane displacement, which is derived below by employing a Fourier transformation technique sharing analogies with the standard technique in classical Cauchy elasticity [20]. The field equation in this case can then be written in the following form (where the exponential term has been factored out):
4.23
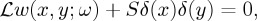
where *δ*(⋅) denotes the Dirac delta distribution and the differential operator 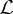 is defined as
4.24
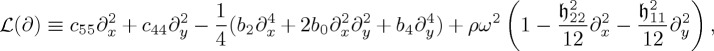
with 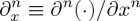. An exact solution to equation (4.23) is obtained by employing the double exponential Fourier transform. The direct and inverse double Fourier transforms of a field *f*(*x*,*y*) are defined as
4.25
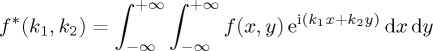
and
4.26
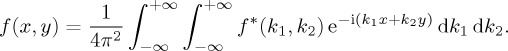
Applying the direct double Fourier transform (4.25) to the field equation (4.23) and performing the inversion yields the out-of-plane displacement in the form
4.27
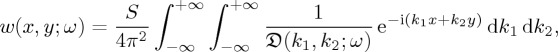
where
4.28
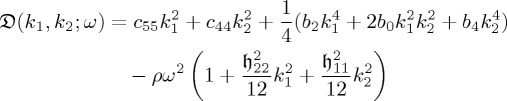
is the characteristic polynomial, related to the 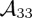 component of the acoustic tensor through 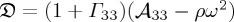. Note that when the (WP) condition holds, 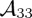 is strictly positive, which, accordingly, implies that the characteristic polynomial 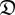 has always real roots for any given frequency. Therefore, the (WP) condition plays the major role for finding the infinite-body Green’s function.

In the simple case of classical elasticity, the characteristic polynomial and the out-of-plane displacement reduce, respectively, to
4.29
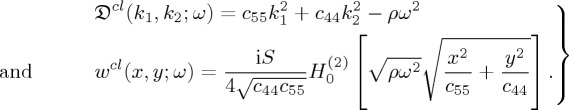
Here 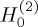 is the Hankel function of the second kind, which, recalling that the time dependence is of the form 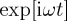, represents *outward*-propagating SH waves.

For the evaluation of the inversion integral in equation (4.27), the integrand is factored by finding the roots of the characteristic quartic polynomial (4.28). For a fixed value of the transformed variable *k*_1_ 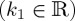, the four roots of 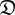 can be written in the following way:
4.30
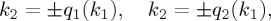
where
4.31
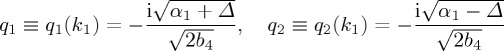
with
4.32
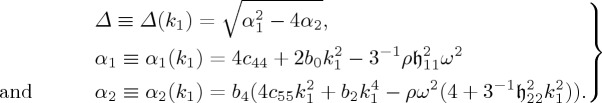
Depending on the values of the transformed variable *k*_1_ and the values of the material parameters, the roots of the characteristic polynomial in equation (4.30) can be: (i) four conjugate imaginary, (ii) two conjugate imaginary and two real, (iii) four complex conjugates and (iv) four real roots. Note that, in all cases, Im[*q*_1,2_]≤0, 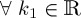. Moreover, if Im[*q*_1,2_]=0, then Re[*q*_1,2_]>0.

The characteristic polynomial can now be written as
4.33
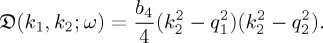
Applying the residue theorem in conjunction with Jordan’s lemma, the integration with respect to *k*_2_ in equation (4.23) yields a summation of residues of poles at *k*_2_=*q*_1_ and *k*_2_=*q*_2_ when *y*>0, or at *k*_2_=−*q*_1_ and *k*_2_=−*q*_2_ when *y*<0. In particular, for *y*>0, the original integration path running along the real axis is replaced by a closed contour taken in the lower *k*_2_-plane, so that the integrand is decaying as 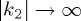. It should be noted that in the cases (ii) or (iv), where two or four roots are real, respectively, the Sommerfeld radiation condition, in view also of equations (4.14) and (4.26), dictates that the closed contour should include the positive real poles when *y*>0 [18]. The following result can then be derived:
4.34
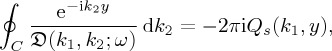
with
4.35
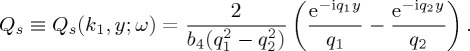
Further, noting that *q*_1_(*k*_1_) and *q*_2_(*k*_1_) are even functions of their argument and by taking also into account equation (4.34), the integral in equation (4.27) can be evaluated as
4.36
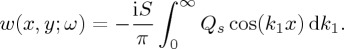
The function *Q*_*s*_ has the following asymptotic properties: (i) *Q*_*s*_=*O*(1) as 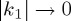 and (ii) 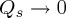 as 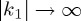. Employing the Abel–Tauber theorem and results from the theory of generalized functions [21], it can be readily shown that property (i) implies that the displacement *w* at infinity (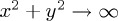) vanishes, as in the classical elastodynamic theory. However, property (ii) suggests that the displacement at the point of application of the load is *finite*, so that the logarithmic singularity (cf. equation (4.29)_2_) of the classical elastodynamic theory is eliminated when Cosserat effects are introduced. An analogous result was obtained in the static antiplane case for a constrained anisotropic Cosserat material [5]. Furthermore, it is noted that the function *Q*_*s*_(*k*_1_,*y*) has square-root (integrable) singularities at the points 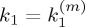, at which 
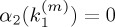
 and 
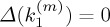
, with 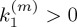. In the light of the above, the integral in equation (4.36) is convergent and can be evaluated numerically taking into account its oscillatory character.

A final comment pertains to the special cases (i) *b*_2_=0 and (ii) 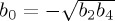, where (E) is lost at the (EI/P) or (EC/H) boundaries, respectively (cf. equations (4.17)). In these cases (see also §4c), the (WP) condition (4.21) still holds, so that the Green’s function (4.23) maintains the same qualitative characteristics as in the case of a regular material far from loss of (E). In fact, it is remarkable that, even in the case of (E) loss, the displacement remains *bounded* everywhere in a constrained Cosserat solid under antiplane deformations. This finding is in marked contrast with the classical elasticity situation, where the loss of (E) implies also loss of (WP) (i.e. the classical acoustic tensor is no longer positive definite) and hence the Green’s function can only be defined in the sense of distributions (cf. equation (5.1)).

In the following, the Green’s function will be used as a perturbing agent to examine the time-harmonic mechanical properties of a series of Cosserat materials with extreme orthotropy. To characterize this orthotropy, it is expedient to introduce the dimensionless parameters
4.37
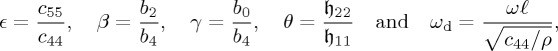
where *ϵ* measures the degree of Cauchy anisotropy, (*β*,*γ*) the degree of couple-stress anisotropy, *θ* the degree of microinertia anisotropy and *ω*_d_ denotes the frequency made dimensionless through division by the classical shear-wave velocity in the *y*-direction (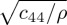) and multiplication by the characteristic material length ℓ. This length ℓ is introduced in the constitutive equations through the relation *b*_4_=4*c*_44_ℓ^2^. The ratio of the characteristic material length ℓ to the microinertia length 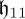 is defined as 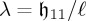. In all cases, it is assumed that *b*_4_>0 and *c*_44_>0. Finally, it is remarked that Cosserat isotropy is recovered when *ϵ*=*β*=*γ*=*θ*=1.

## 5. Dynamic folding of an elastic constrained Cosserat continuum

A constrained Cosserat solid close to loss of ellipticity (E) exhibits extreme orthotropic properties and is prone to folding instabilities. Following [5], folding is here revealed through a perturbation of the material by a concentrated time-harmonic force, in the way introduced for (non-polar) elastic prestressed materials [22,23]. During folding, the displacement gradient suffers a *finite* jump across a discontinuity line, whereas the displacement field becomes locally a continuous, piecewise-smooth, function exhibiting a cusp along the discontinuity line. It should be remarked that the applied concentrated force (Green’s function) is to be understood as a perturbation demonstrating that the material tends towards the state of folding, when subject to a generic mechanical action. In this way, folding emerges as a material instability for a constrained Cosserat anisotropic material, similarly to the situation occurring when a shear band forms in an elastoplastic material [23]. It is worth noting that the instability phenomenon of folding cannot be captured within the context of the classical elasticity theory.

**Figure 1.**
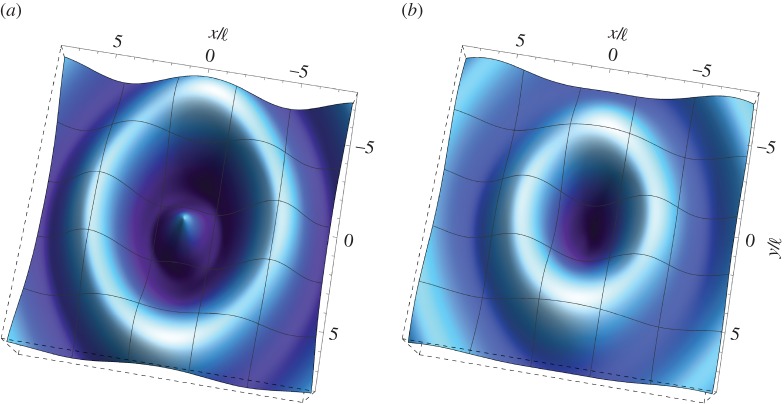
Dynamic response of an orthotropic material without microinertia far from loss of (E). Real (*a*) and imaginary (*b*) parts of the dimensionless out-of-plane displacement 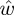 as produced by an antiplane concentrated time-harmonic force at the dimensionless frequency *ω*_d_=1. The material is characterized by *β*=1/2, *γ*=1/4 and *ϵ*=1/4. Note that the displacement is bounded everywhere. (Online version in colour.)

To facilitate comparisons, an illustrative example of a ‘non-extreme’ constrained Cosserat material is presented in figure 1. In particular, the dimensionless out-of-plane displacement 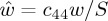 is plotted for an orthotropic Cosserat material without microinertia (*θ*=λ=0), far from the (E) boundary (*ϵ*=1/4, *β*=1/2, *γ*=1/4), as produced by a concentrated time-harmonic antiplane force *S* (acting at the origin of the axes), with frequency *ω*_d_=1. The real and imaginary parts of the Green’s function are shown separately (figure 1*a* and *b*, respectively). It is observed that, in contrast with the result of the classical elastodynamic theory (cf. equation (4.29)_2_), the displacement is bounded at the point of application of the concentrated force with 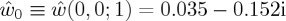 (this value provides the scale of the plots).

It is interesting to note that, for a constrained Cosserat material with null shear modulus in the *x*-direction, *c*_55_=0 (or equivalently *ϵ*=0), neither the (E) nor the (WP) conditions are violated. Accordingly, no localization or any kind of instability are observed in the Cosserat material. On the other hand, for a Cauchy material with *c*_55_=0, both the (E) and (WP) conditions are lost and the Green’s function can only be interpreted in the sense of distributions. In this case, the inversion integral in (4.27), in conjunction with equation (4.29)_1_, yields an out-of-plane displacement of the form
5.1
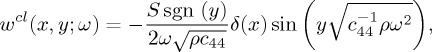
which shows a Dirac-type localization along the discontinuity line *x*=0.

### (a) Dynamics of folding patterns in a medium without microinertia

In the case of an orthotropic constrained Cosserat material, folding occurs at loss of (E) either on the (EI/P) boundary (where *β*=0 and *γ*>0 or, equivalently, *b*_2_=0 and *b*_0_>0) or on the (EC/H) boundary (where 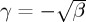 and *β*>0 or, equivalently, *b*_2_>0 and 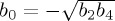) [4]. In the former case, a *single* fold (crease) appears along the discontinuity line *x*=0, whereas in the latter case folding emerges in a *cross*-type geometry, with two inclined discontinuity lines. The inclination *ϕ* of the discontinuity lines with respect to the *y*-axis is given by the condition 
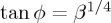
 [4]. Note that, in both the above cases, the Green’s function (4.27) is well defined under the hypothesis that the (WP) condition (4.21) holds.

**Figure 2.**
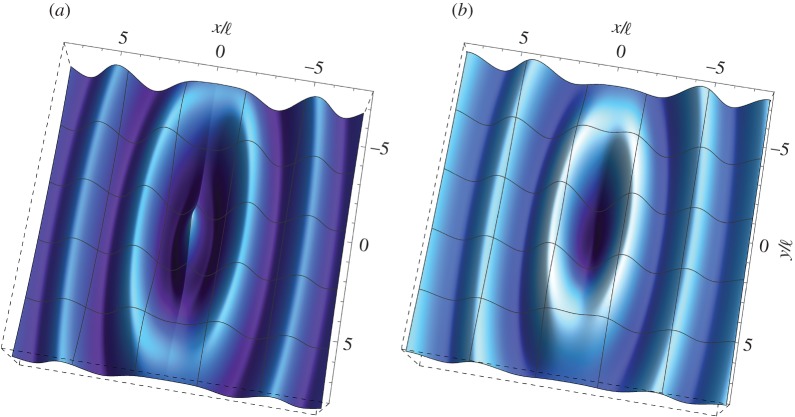
Dynamic response of an extreme orthotropic material without microinertia at the (EI/P) boundary for loss of (E). Real (*a*) and imaginary (*b*) parts of the dimensionless out-of-plane displacement 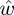 as produced by an antiplane concentrated time-harmonic force at the dimensionless frequency *ω*_d_=1. The material is characterized by *β*=0, *γ*=1/4 and *ϵ*=1/4. Note that the real part (*a*) of the displacement 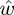 exhibits single folding along the discontinuity line *x*=0. (Online version in colour.)

Figure 2 illustrates the formation of a localized single folding at the frequency *ω*_d_=1 for a Cosserat material with null microinertia (λ=*θ*=0), characterized by *β*=0, *γ*=1/4 and *ϵ*=1/4. The displacement at the point of application of the load is 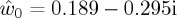, which provides the scale of the plots. It is observed that only the real part of the solution exhibits folding, whereas the imaginary part remains smooth. As shown in §5b(iii), the displacement gradient ∂*w*/∂*x* displays a finite jump *across* the discontinuity line *x*=0, thus showing that the solution suffers a weak shock.

**Figure 3.**
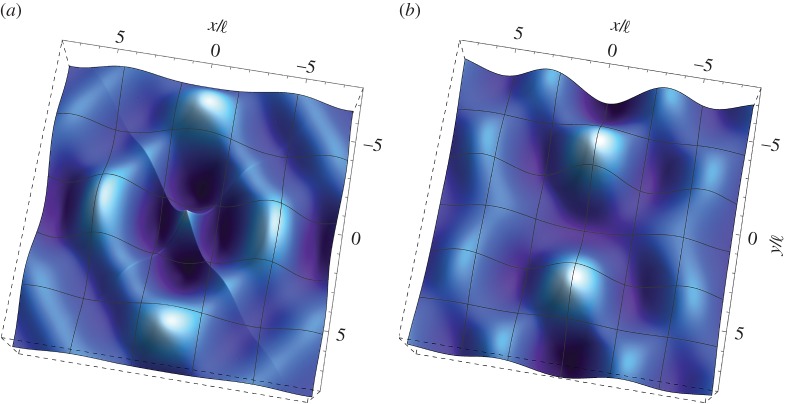
Dynamic response of an extreme orthotropic material without microinertia at the (EC/H) boundary for loss of (E). Real (*a*) and imaginary (*b*) parts of the dimensionless out-of-plane displacement 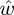 as produced by an antiplane concentrated time-harmonic force at the dimensionless frequency *ω*_d_=1. The material is characterized by *β*=1/2, 
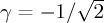
 and *ϵ*=1/4. Note that the real part (*a*) of the displacement 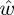 exhibits a cross folding along two discontinuity lines inclined at *ϕ*=40° with respect to the *y*-axis. (Online version in colour.)

Figure 3 shows the formation of localized cross folding for a Cosserat material without microinertia (λ=*θ*=0), characterized by *β*=1/2, 
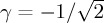
 and *ϵ*=1/4. The inclination of the discontinuity lines is *ϕ*=40° and 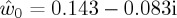. Note that, as in the single folding case, only the real part of the solution exhibits folding.

**Figure 4.**
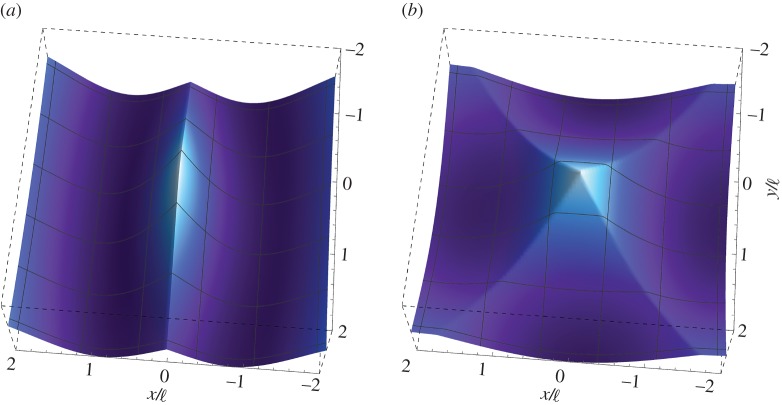
Details of figures 3*a* and 4*a* showing the single (*a*) and cross (*b*) folding patterns emerging in extreme orthotropic constrained Cosserat continua with null microinertia near loss of (E). (Online version in colour.)

The formation of single and cross folding patterns in the constrained Cosserat material is more clearly depicted in figure 4, where the real part of the out-of-plane dimensionless displacement is plotted in a region close to the point of application of the concentrated force. It is observed that in both cases the folding angle decays away from the origin.

### (b) The role of microinertia

The role of microinertia is investigated in this section, as connected to the formation of folding patterns. An inspection of equation (4.15) shows that the microinertia lengths 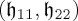 are related to the (lower) second-order spatial derivatives of the out-of-plane displacement. In fact, for fixed values of the frequency *ω*, the terms 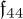 and 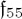 may change sign according to the magnitude of the microinertia lengths. As will be subsequently shown, the nature of the solution depends indeed upon the sign of these quantities. In what follows, the cases of single and cross folding will be treated separately.

#### (i) Single folding

**Figure 5.**
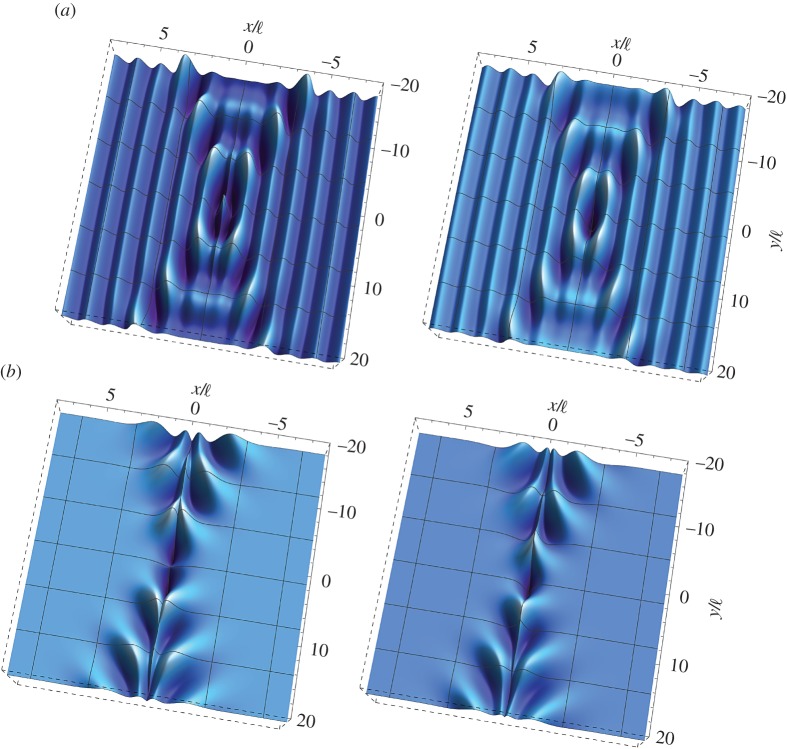
Dynamic response of an extreme orthotropic material with microinertia at the (EI/P) boundary for loss of (E). Real (left) and imaginary (right) parts of the dimensionless out-of-plane displacement 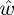 as produced by an antiplane concentrated time-harmonic force at the dimensionless frequency *ω*_d_=1. The material is characterized by *β*=0, *γ*=1/4, *ϵ*=1/4, λ=1 and (*a*) *θ*=0.9θ* and (*b*) *θ*=1.1θ*. Note that for case (*a*) the disturbance degenerates into waves propagating only parallel to the folding line (*x*=0), whereas for case (*b*) the disturbance rapidly decays in the *x*-direction, but does not along the discontinuity line *x*=0, thus showing an example of a folding wave. (Online version in colour.)

For single folding emerging at the (EI/P) boundary of loss of (E), two special cases are considered highlighting the effects of microinertia, namely (i) 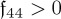 and 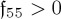, and (ii) 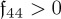 and 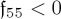. In particular, figure 5 shows the behaviour of the dimensionless out-of-plane displacement 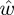 for a concentrated time-harmonic force placed at *ω*_d_=1. The extreme orthotropic Cosserat material is characterized by parameters *β*=0, *γ*=1/4, *ϵ*=1/4, λ=1 and (*a*) *θ*=0.9θ* (so that 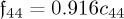, 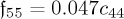) and (*b*) *θ*=1.1θ* (so that 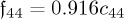, 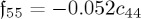). Note that the special value 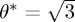 corresponds to the case where 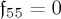 at *ω*_d_=1.

It is observed from figure 5*a* that, as the microinertia parameter 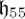 increases, the wavelength of the disturbance decreases significantly when compared with a Cosserat medium without microinertia (figure 2). Moreover, the wavefronts now become parallel to the discontinuity line *x*=0. Further increase of the microinertia 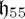 results in 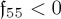 and the response to the perturbation changes qualitatively. Indeed, it is shown in figure 5*b* that the disturbance corresponds to a mode of rapidly decaying oscillations in the direction normal to the discontinuity line (***n***=(±1,0)). As pointed out in §4b, the change of sign in the term 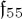 corresponds to passing from a tensile to a compressive prestress in the *y*-direction in an orthotropic plate with microinertia. On the other hand, in the direction parallel to the discontinuity line, the disturbance oscillates with a slowly decaying amplitude (see also figure 7*b*, blue curve) confined in a small zone 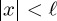, giving rise to a ‘folding wave’ (see the discussion below). It is worth noting that, different from the case in figure 5*a*, both the real and imaginary parts of the solution evidence folding.

#### (ii) Cross folding

**Figure 6.**
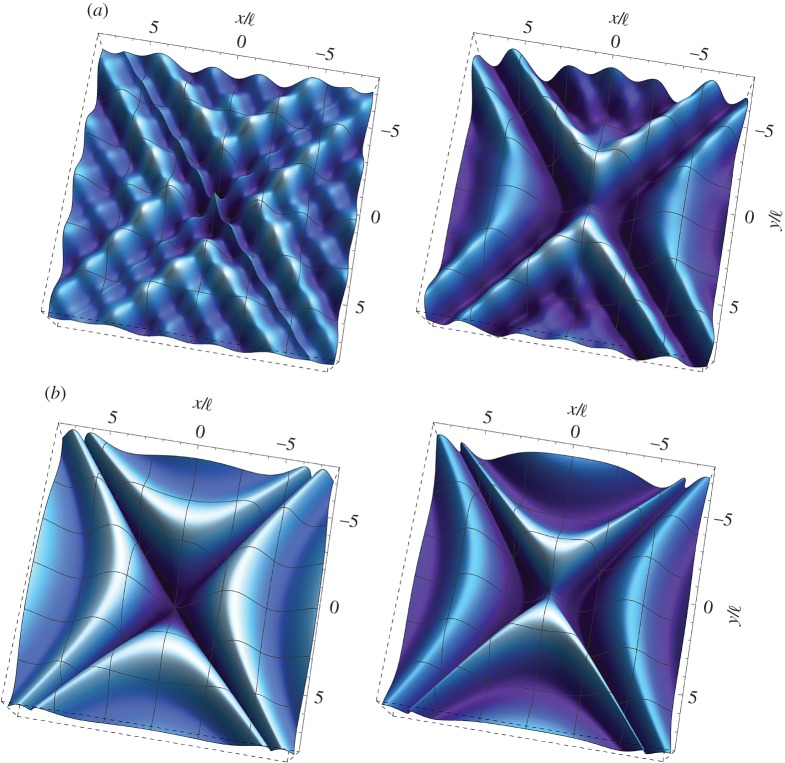
Dynamic response of an extreme orthotropic material with microinertia at the (EC/H) boundary for loss of (E). Real (left) and imaginary (right) parts of the dimensionless out-of-plane displacement 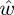 as produced by an antiplane concentrated time-harmonic force at frequency *ω*_d_=1. The material is characterized by the parameters *β*=1/2, 
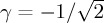
, *ϵ*=1/4, *θ*=1/2 and (*a*) λ=0.9λ* and (*b*) λ=1.1λ*. (Online version in colour.)

For cross folding occurring at the (EC/H) boundary of loss of (E), two particular cases are considered, namely (i) 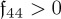 and 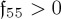, and (ii) 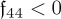 and 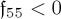. Figure 6 shows the behaviour of dimensionless out-of-plane displacement 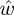 for a concentrated time-harmonic force at *ω*_d_=1. The orthotropic Cosserat material is characterized by the parameters *β*=1/4, 
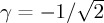
, *ϵ*=1/4, *θ*=0.5 and (*a*) λ=0.9λ* (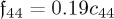, 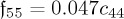) and (*b*) λ=1.1λ* (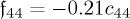, 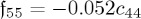). Note that the special value 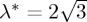 corresponds to 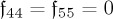 at *ω*_d_=1.

As in the case of single folding, it is observed that the wavelength of the disturbance decreases significantly as 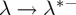 (approaching the limit from below) compared with the respective result for a Cosserat medium with null microinertia (figure 3). In addition, the disturbance is produced by the superposition of two wavefronts parallel to the discontinuity lines inclined at *ϕ*=40°, which propagate with decreasing amplitude. For λ>λ*, the disturbance becomes confined in a zone close to the lines of discontinuity and decays quickly away from them. In this case, both the real and imaginary parts of the solution display folding.

#### (iii) Folding waves

To investigate further the nature of folding instabilities and the role of microinertia, the conditions under which the formation of a discontinuity line becomes possible at the (EI/P) boundary of ellipticity loss are now studied (the general conditions for a three-dimensional anisotropic body were obtained in [4]) with a view towards examining the possibility of a propagating folding wave (as shown in figure 5*b*),

From equilibrium considerations and imposing continuity for the out-of-plane displacement across a surface defined by the unit normal ***n***=(±1,0), the following relations are derived:
5.2
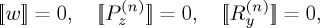
where [[ ]] denotes the jump of the enclosed quantity across the relevant surface. The expression for the tractions in the antiplane case considered here can be derived from the general definitions (2.6). Note that, employing Hadamard’s lemma, equation (5.2)_1_ implies [[∂_*y*_*w*]]=∂_*y*_[[*w*]]=0.

Using the kinematical conditions (4.2) in conjunction with the constitutive equations (4.3) and (4.4), and bearing in mind that *b*_2_=0 (*b*_0_>0) for the (EI/P) case, equation (5.2)_3_ is identically satisfied, while
5.3
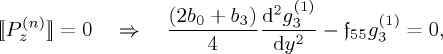
where the discontinuity vector 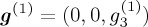 with 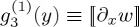 defines the unknown jump in the normal (to the discontinuity line *x*=0) derivative of the out-of-plane displacement. The folding angle *ψ*(*y*) can then be defined through the equation 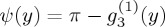.

Assuming that the (WP) condition (4.21) holds, the second-order ordinary differential equation (5.3) admits the general solution
5.4
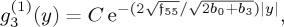
where *C* is a non-zero constant.

The above result shows that, when (E) is lost but the (WP) condition still holds, a *non-zero* discontinuity vector 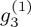 becomes possible. It is apparent from (5.4) that the behaviour of the jump 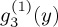 and of the folding angle *ψ*(*y*) depend on the sign of the term 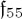. In particular, for 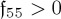, the jump becomes exponentially decaying along the discontinuity line and 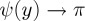 as 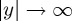 (corresponding to the absence of folding). An analogous situation was encountered in the quasi-static case [5]. However, for 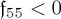, the jump is *propagating* without any decay, which implies that a *folding wave* propagates along the discontinuity line *x*=0.

It is worth noting that the expression (5.4), describing the variation of 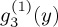, involves the Cosserat modulus *b*_3_ (the secondary bending stiffness). However, this bending stiffness is not involved in the infinite-body Green’s function, equation (4.27), which depends on the parameter *b*_0_ (with *b*_0_=*b*_1_−*b*_3_). In fact, although the relations for continuity of tractions (5.2) hold for any value of the Cosserat modulus *b*_3_, it is apparent from (4.6)_4_ that, in order for 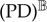 and (E) to be lost simultaneously, the parameter *b*_3_ must be set to zero, *b*_3_=0, so that the emerging discontinuity line becomes *admissible* (see also [5]).

**Figure 7.**
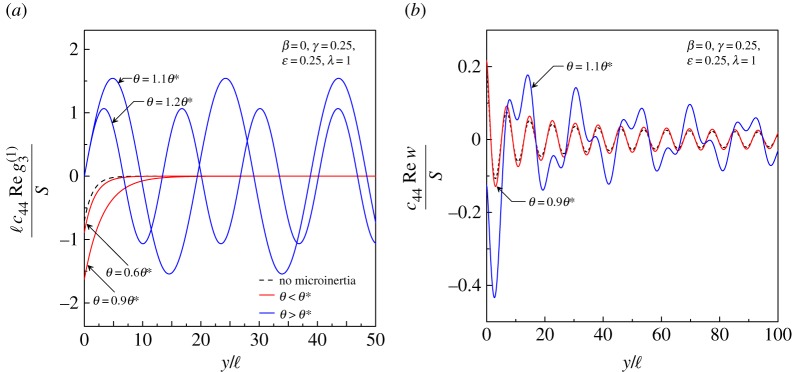
(*a*) Variation of the real part of the normalized jump 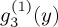 along the discontinuity line. The jump becomes exponentially decaying for *θ*<θ*, whereas for *θ*>θ* it propagates along the discontinuity line forming a folding wave. (*b*) Variation of the real part of the dimensionless displacement along the discontinuity line. (Online version in colour.)

Additional insight into the circumstances just described is provided by figure 7, where (*a*) the real part of the normalized jump 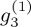 and (*b*) the real part of the dimensionless out-of-plane displacement are depicted along the folding line *x*=0 for a Cosserat material at the (EI/P) boundary for loss of (E) with *β*=0, *γ*=1/4, *ϵ*=1/4 and λ=1. It is observed that, when the microinertia is null (dashed curve) or when *θ*<θ* (red curves in figure 7*a*), the jump, according to equation (5.4), is a real-valued exponentially decaying function of *y*. On the other hand, for *θ*>θ* (blue curves in figure 7*a*), 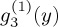 propagates along the discontinuity line with a constant amplitude which depends on the parameter *θ*. In all cases, the out-of-plane displacement decreases away from the origin (figure 7*b*). It is interesting to note that, for *θ*>θ*, the pulse is distorted during propagation, implying that the disturbance becomes highly dispersive (figure 7*b*, blue curve).

Finally, it should be noted that the above results and observations can be readily extended to the case where (E) is lost at the (EC/H) boundary in the same spirit as [5].

## 6. Conclusion

The conditions for wave propagation have been explored for orthotropic constrained Cosserat elastic solids in the presence of microinertia. The acoustic tensor and the conditions for loss of ellipticity have been derived, together with a new infinite-body Green’s function for time-harmonic vibrations. Employed as an agent perturbing an infinite medium, the Green’s function has revealed the interplay between dynamics and folding mechanisms in Cosserat materials. In particular, the effect of microinertia has been proved to be connected with the propagation of special disturbances, called ‘folding waves’.

## Footnotes

1 When a dynamic motion is considered in a non-inertial frame, the presence of inertia and microinertia in the equation of motion and in the boundary conditions may violate the assumption of Euclidean objectivity. As Jaunzemis [9], p. 233 points out, the issue of objectivity, in such cases, can be circumvented by introducing an objective generalized (effective) body force and an objective generalized body couple. The generalized body force  (cf. (2.2)) is defined as the difference of the standard body force and the inertia term (related to the acceleration), whereas the generalized body couple  (cf. (2.3)) is defined as the difference of the standard body couple and the microinertia term (related to the acceleration gradient in the constrained Cosserat theory). The generalized body force and body couple are assumed to be objective although their constituents are not (see also [10]). It is remarked that throughout this study an inertial frame is employed.
